# Nutritional Complications After Laparoscopic Roux-en-Y Gastric Bypass and One-Anastomosis Gastric Bypass: A Comparative Systematic Review and Meta-Analysis

**DOI:** 10.7759/cureus.21114

**Published:** 2022-01-11

**Authors:** Mohamed Tourky, Mohamed Issa, Mohamed A Salman, Ahmed Salman, Hossam El-Din Shaaban, Ahmed Safina, Abd Al-Kareem Elias, Ahmed Elewa, Khaled Noureldin, Ahmed Abdelrahman Mahmoud, Ahmed Dorra, Mohamed Farah, Mahmoud Gebril, Mujahid Gasemelseed Fadlallah Elhaj, Hesham Barbary

**Affiliations:** 1 General Surgery, Great Western Hospital, NHS Foundation Trust, Swindon, GBR; 2 Surgery, Wirral University Teaching Hospital NHS Foundation Trust, Birkenhead, GBR; 3 Surgery, Prince Charles Hospital, Myrther Tydfil, GBR; 4 Surgery, KasrAlainy School of Medicine, Cairo University, Cairo, EGY; 5 Internal Medicine, KasrAlainy School of Medicine, Cairo University, Cairo, EGY; 6 Gastroenterology and Hepatology, National Hepatology and Tropical Medicine Research Institute, Cairo, EGY; 7 General Surgery, KasrAlainy School of Medicine, Cairo University, Cairo, EGY; 8 General Surgery, Faculty of Medicine, Al-Azhar University, Assuit Branch, Kafr El-Sheikh, EGY; 9 General Laparoscopic and Hepato-Pancreatico-Biliary (HBP) Surgery, National Hepatology and Tropical Medicine Research Institute, Cairo, EGY; 10 Colorectal Surgery, Southend University Hospital, Mid and South Essex NHS Foundation Trust, Essex, GBR; 11 Surgery, Leighton Hospital, Mid Cheshire Hospitals NHS Foundation Trust, Crewe, GBR; 12 Urology, Sunderland Royal Hospital, South Tyneside and Sunderland NHS Foundation Trust, Sunderland, GBR; 13 General and Laparoscopic Surgery, Al Qabary Specialized Oncology Center, Alexandria, EGY; 14 General Surgery, Saudi German Hospital, Jeddah, SAU; 15 General and Laparoscopic Surgery, El Zaitoun Specialized Hospital, Cairo, EGY

**Keywords:** one-anastomosis gastric bypass, comparative, hypoalbuminemia, malnutrition, roux-en-y, gastric bypass

## Abstract

A systematic review and meta-analysis were carried out involving studies that compared the nutritional complications of Roux-en-Y gastric bypass (RYGB) and one-anastomosis gastric bypass (OAGB); these included the incidence of malnutrition as well as deficiencies of other nutritional elements, such as total protein, albumin, calcium and iron. A comprehensive search strategy was implemented in PubMed, Embase, and the Cochrane Library. Effect sizes included the pooled odds ratios (ORs) and 95% confidence intervals (95% CIs), as well as mean differences (MDs) and 95% CIs of the percentage total weight loss (%TWL) and excess weight loss percentage (%EWL).

Thirteen studies were included (12,964 patients, 66.27% females, 53.82% underwent OAGB). At the longest follow-up period (≥3 years), OAGB was associated with significantly higher %TWL (MD=5.41%, 95%CI, 1.52 to 9.29) and %EWL (MD=13.81%, 95%CI, 9.60 to 18.02) compared to RYGB. However, OAGB procedures were associated with malnutrition (OR=3.00, 95%CI, 1.68 to 5.36, p<0.0001), hypoalbuminemia (OR=2.38, 95%CI, 1.65 to 3.43, p<0.0001), hypoproteinemia (OR=1.85, 95%CI, 1.09 to 3.14, p=0.022), anemia (OR=1.38, 95%CI, 1.08 to 1.77, p=0.011), and hypocalcemia (OR=1.78, 95%CI, 1.01 to 3.12, p=0.046). On subgroup analyses, the proportions of anemia and hypoalbuminemia remained significantly higher at longer follow-up periods and in studies published in Asia.

Despite the favorable weight loss profile, the unfavorable nutritional consequences of OAGB merits further investigations to explore the malabsorptive element, ethnic variation, and the role of biliopancreatic limb length.

## Introduction and background

Introduction

Obesity and its related comorbidities have become an important health priority due to their effects on life quality and expectancy. The prevalence of obesity has dramatically increased worldwide, with more than 650 million obese adults and 2.5 million obesity-related deaths as of 2016 [1]. With the projected increase in obesity prevalence over the next decades [2,3], it is necessary to adopt effective managemental approaches via lifestyle, medical and surgical interventions. In particular, bariatric surgery has proven effective to achieve adequate weight loss, particularly among patients with morbid obesity [4].

Roux-en-Y gastric bypass (RYGB) is a restrictive-malabsorptive procedure that is superior to non-surgical treatment in terms of weight loss outcomes and resolution of co-morbidities [5,6]. It was first introduced by Mason in 1966 [7] and the first laparoscopic procedure was performed in 1994 by Alan Wittgrove [8]. With the exponential increase in the number of bariatric surgeries since the early 2000s, RYGB has been considered the gold standard procedure for the management of morbidly obese patients [9]. The procedure involves creating a small gastric pouch connected to a roux limb (gastrojejunostomy) and an additional jejunojejunostomy is created at the level of the proximal jejunum. Laparoscopic RYGB is one of the most technically demanding laparoscopic techniques and requires appropriate surgical skills [10].

In 2001, Rutledge [11] proposed a modified, less technically-demanding technique, namely mini-gastric bypass, which was subsequently modified to one-anastomosis gastric bypass (OAGB) [12]. It consists of the formation of a gastrojejunal anastomosis between a long, narrow gastric pouch (restrictive part) and a jejunal omega loop (malabsorptive part) [13]. OAGB surgery has gained popularity over the past few years, and it has shown comparable efficacy and safety outcomes to those of the RYGB technique [14]. 

However, despite their clinical benefits, RYGB and OAGB procedures might be associated with a number of surgical and gastrointestinal complications, of which malabsorptive complications deserve considerable attention. These include anemia, deficiencies of vitamins and minerals, protein malnutrition, and abnormalities of bone metabolism [15,16]. Relevant data based on randomized clinical trials of nutritional deficiencies, and complications are scarce [17,18] and, to the best of our knowledge, no comprehensive systematic reviews have been conducted as yet to compare the nutritional outcomes of RYGB and OAGB. Considering the accumulated evidence so far, we conducted a systematic review and meta-analysis of studies that compared the nutritional complications of RYGB and OAGB.

## Review

Methods

Eligibility Criteria

The present study was designated based on the guidelines of the Preferred Reporting Items for Systematic Reviews and Meta-analyses (PRISMA) statement [19]. Eligible studies should have compared the incidence of at least one postoperative nutritional complication at follow-up (at ≥ 1 year) after RYGB and OAGB procedures. RCTs, prospective cohort investigations, and retrospective studies written in English were eligible. Systematic reviews, narrative reviews, and case reports were excluded.

Types of Outcomes Measures

The primary outcomes included the frequencies of patients with nutritional complications at the most recent follow-up timepoint. These complications included anemia, hypoalbuminemia, hypocalcemia, hypoproteinemia, as well as serum deficiencies of vitamin D, vitamin B12, zinc, iron, and ferritin. Percentage total weight loss (%TWL) and excess weight loss (%EWL) at follow-up were considered secondary outcomes.

Search Strategy

The search strategy was carried out on three academic databases, including PubMed, Embase, and the Cochrane Library up to October 25, 2021. No limitations were set to the date of publication or study design. A relevant search strategy was developed using specific search terms, which were combined using the predefined Boolean operators (AND, OR, and NOT). An example of the used strategy is demonstrated in Appendix 1.

Study Selection and Data Collection

Screening of the obtained records across the academic databases was performed by two independent authors. A reference list was uploaded from each library to a dedicated software (EndNote X9, released 2013, Clarivate™, Philadelphia, Pennsylvania, United States) to detect and remove duplicate records. The bibliographies of screened articles were additionally searched for potentially eligible studies. The full-text versions of eligible articles were downloaded and checked against the inclusion/exclusion criteria. A specific spreadsheet was created on Microsoft Excel 2016 (Microsoft® Corp., Redmond, Washington, United States) for data collection. The extracted data included the following domains: (1) study characteristics, including the last name of the first author, study setting (country), date of publication, study design, and the latest follow-up timepoint; (2) patients’ characteristics at baseline, including the gender, age, body mass index; (3) procedural characteristics, including the number of patients who underwent RYGB and OAGB and the length of the alimentary (roux) limb in RYGB procedures and biliopancreatic limbs in RYGB and OAGB; (4) outcome variables: the frequencies of patients with nutritional complications. For the primary and secondary outcomes, data were collected from the most recent follow-up visit for each study.

Quality Assessment

The recommended Cochrane risk of bias tool [20] was used to assess the methodological quality of RCTs. Such a tool measures the risk of bias in selected quality indicators, including random sequence generation, allocation concealment, attrition bias, and blinding. Regarding non-randomized studies, the Newcastle-Ottawa Scale (NOS) [21] was utilized to explore the methodological quality in terms of participants’ selection, comparability, and outcomes. A NOS score was calculated by summing up the number of stars allocated to the selected items. Studies with a NOS score of 4-6 and ≥ 7 were considered of medium or high quality, respectively.

Statistical Analysis

The GetData™ Graph Digitizer version 2.26 (GetData Pty Ltd, Kogarah, New South Wales, Australia) was used to extract numerical outcomes from visually-depicted data. During data extraction, SDs were derived from confidence intervals (CIs) using the standard methods of the Cochrane handbook for systematic reviews of interventions [22]. In addition, means and SDs were estimated from the reported medians and interquartile ranges as described earlier [23]. The differences in frequencies of nutritional complications between RYGB and OAGB were analyzed by calculating the pooled odds ratios (ORs) and their respective 95%CIs. Furthermore, the pooled differences in %TWL and %EWL were estimated using mean differences (MDs) and 95%CIs. Statistical heterogeneity between studies was explored using an Identify and Interpret (I^2^) method, and it was considered statistically significant at I2>50%. Random-effects were applied on models with significant heterogeneity; otherwise, fixed-effects models were used. Subgroup analysis was performed based on sample size, study location, follow-up periods, and study design. Based on the sample size, studies were categorized into small (n< 500), medium (n = 500-1500) and large-sized studies. Assessment of publication bias was performed by producing funnel plots and interpreting the outcomes of Egger’s test. The meta-analysis was performed using the metacont and metabin packages in R (RStudio 2021.09.2+382, RStudio Corp., Boston, Massachusetts, United States).

Results

Results of the Search Process

**Figure 1:**
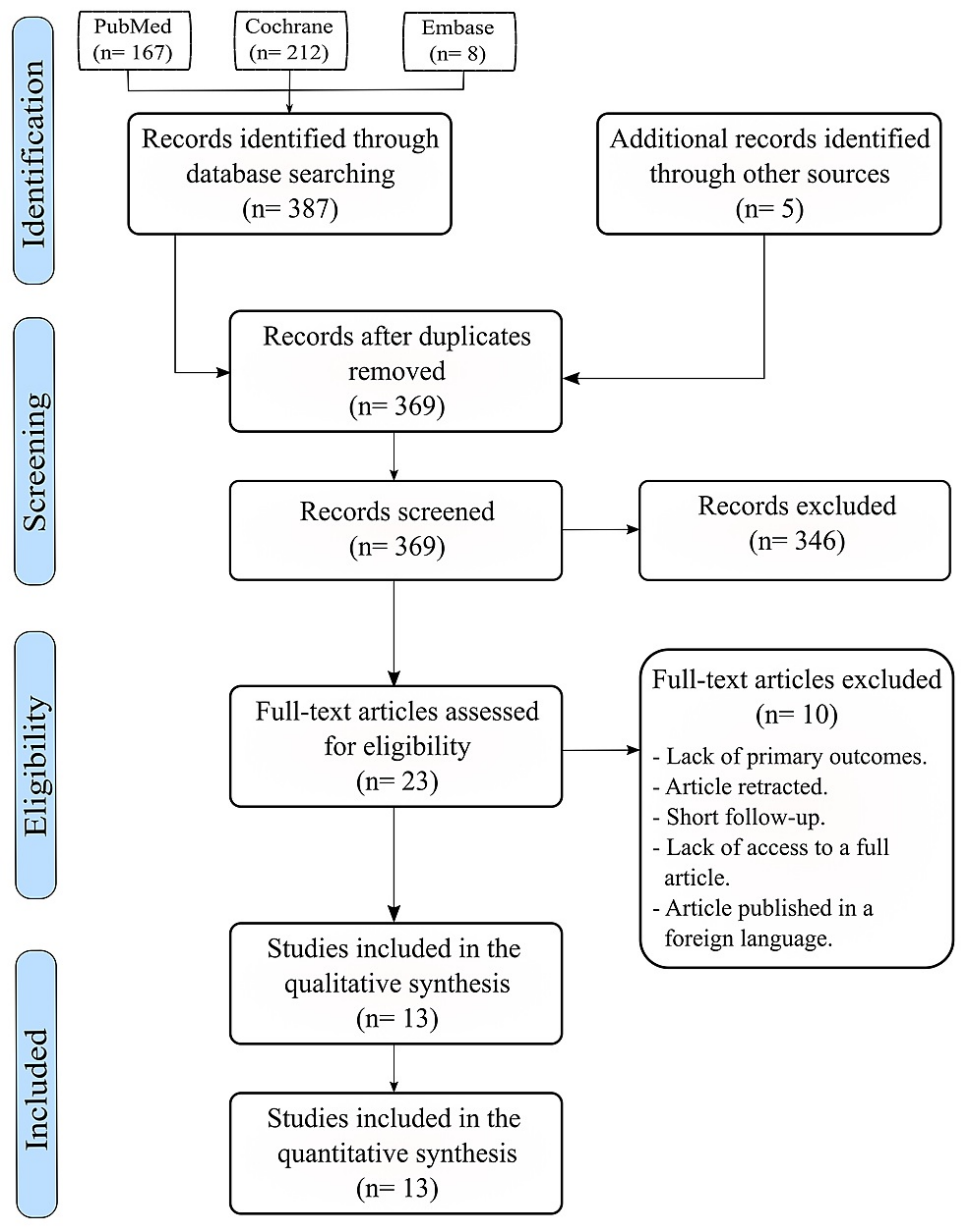
Studies included in the systematic review and meta-analysis

Initially, we identified a total of 392 records across all databases, of which 23 duplicate records were excluded. Out of the remaining records (n=369), the full article version of 23 articles were thoroughly assessed for eligibility. However, 10 articles were excluded due to lack of primary outcomes [24-28], article retraction [29], short follow-up periods (six months) [30,31], lack of access to a full article [32], and an article published in a non-English language [33]. Therefore, 13 studies were formally included in our systematic review and meta-analysis (Figure 1).

Characteristics of the Included Studies

Study-related characteristics are demonstrated in Table 1. Out of the included studies, there was one RCT [17], while the remaining studies employed a retrospective investigation of patients’ records. Eight studies were published in Asian countries [34-41], four studies in Europe [17,42-44], and one study in Australia [45]. Follow-up periods ranged between one and 15 years. Regarding patients’ characteristics, 12,964 patients were studied in the included articles, of whom 66.27% were females. Furthermore, 53.82% and 46.18% of patients had undergone OAGB and RYGB procedures, respectively. Other demographic characteristics of patients are listed in Table 2.

**Table 1:** Characteristics of the included studies N: number; Y: years; OAGB: one-anastomosis gastric bypass; BPL: biliopancreatic limb; RYGB : Reux-en-Y gastric bypass; NOS: Newcastle-Ottawa score; RCT: randomized controlled trial

Author, year	Country	Study design	N of bariatric sites	Follow-up period (y)	Length of bowel segments (cm)			NOS
					OAGB BPL	RYGB alimentary (roux) limb	RYGB BPL	
Alkhalifah et al. 2018 [34].	Taiwan	Retrospective	1	15	150–250	150	100	7
Baig et al. 2019 [35].	India	Retrospective	25	5	150-210	70-150	50-125	7
Bhandari et al. 2019 [36].	India	Retrospective	1	5	250	120	80	7
Chen et al. 2019 [37].	Taiwan	Retrospective	1	1	150-400	150-350	100	7
Jammu et al. 2016 [38].	India	Retrospective	1	7	200	75-150	50	7
Khalaj et al. 2020 [39].	Iran	Retrospective	1	1	200	150	50	7
Lee et al. 2012 [40].	Taiwan	Retrospective	1	5	200	120	60	7
Madhok et al. 2018 [42].	UK	Retrospective, case matched	1	2	200	150	50	7
Rheinwalt et al. 2020 [43].	Germany	Retrospective	1	3	200-300	160	80	9
Robert et al. 2019 [17].	France	Open-label RCT (YOMEGA)	9	2	200	150	50	NA
Soheilipour et al. 2021 [41].	Iran	Retrospective	1	1	NA	NA	NA	7
Voglino et al. 2021 [44].	Italy	Retrospective, case matched	1	3	200	150	80	7
Zarshenas et al. 2021 [45].	Australia	Retrospective	1	2	200	100	100	7

**Table 2:** Patient characteristics at baseline N: number; M: male; F: female; OAGB: one-anastomosis gastric bypass; RYGB: Reux-en-Y gastric bypass; T: total number

Author, year	N of patients	Gender (M/F)		Age, years (Mean ± SD)		BMI, kg/m^2^ (Mean ± SD)	
	OAGB/RYGB/T	OAGB	RYGB	OAGB	RYGB	OAGB	RYGB
Alkhalifah et al. 2018 [34].	1731/805/2536	519/1212	232/573	33.80 ± 10.40	35.40 ± 10.10	40.40 ± 7.70	38.50 ± 6.50
Baig et al. 2019 [35].	1194/2965/4159	548/646	1373/1592	43.07 ± 11.42	43.98 ± 11.65	45.08 ± 8.82	44.93 ± 7.91
Bhandari et al. 2019 [36].	90/122/212	60/30	65/57	44.00 ± 10.90	46.40 ± 10.50	46.00 ± 6.90	42.00 ± 6.20
Chen et al. 2019 [37].	1022/377/1399	326/696	109/268	34.40 ± 10.90	35.90 ± 10.60	41.20 ± 7.70	38.60 ± 6.70
Jammu et al. 2016 [38].	473/295/768	140/333	85/210	NA	NA	NA	NA
Khalaj et al. 2020 [39].	272/145/417	41/231	29/116	38.90 ± 10.70	40.20 ± 10.70	46.70 ± 6.40	44.50 ± 5.80
Lee et al. 2012 [40].	1163/494/1657	313/850	132/362	32.30 ± 9.10	33.50 ± 9.30	41.10 ± 6.10	40.50 ± 5.80
Madhok et al. 2018 [42].	200/200/400	61/139	61/139	45.00 ± 11.40	45.00 ± 11.00	49.00 ± 7.30	48.00 ± 6.70
Rheinwalt et al. 2020 [43].	324/288/612	82/242	58/230	42.51 ± 11.36	41.40 ± 10.04	53.75 ± 6.51	44.53 ± 3.65
Robert et al. 2019 [17].	117/117/234	32/85	26/91	44.40 ± 11.40	52.60 ± 10.20	43.80 ± 6.10	43.90 ± 5.10
Soheilipour et al. 2021 [41].	289/94/383	NA	NA	NA	NA	NA	NA
Voglino et al. 2021 [44].	57/57/114	12/45	11/46	41.00 ± 9.89	41.00 ± 8.37	46.30 ± 6.85	46.80 ± 5.55
Zarshenas et al. 2021 [45].	45/28/73	13/32	6/22	52.70 ± 11.30	50.50 ± 9.50	47.10 ± 8.00	42.50 ± 7.30

Regarding the results of quality assessment, retrospective studies had generally high methodological quality (NOS ranged between seven and nine, Table 1). Patient selection was appropriate since patients in both surgical groups were selected from the same population (obese patients), and their data were retrieved from secure hospital records. The authors in only one study have adjusted the analyses for age and other demographic characteristics [43]. Concerning the included RCT [17], selection bias was judged as “low risk” because group randomization was performed using a computer-generated sequence, and patients were allocated using sealed envelopes. However, there was a high risk of performance bias since the blinding of the participants and study personnel was not possible (open-label) due to procedural differences. The risks of attrition bias (incomplete outcome data) and reporting bias (selective reporting) were considered “low” because all the outcomes were adequately reported with no potential effects on the results due to withdrawals.

Weight Loss

**Figure 2:**
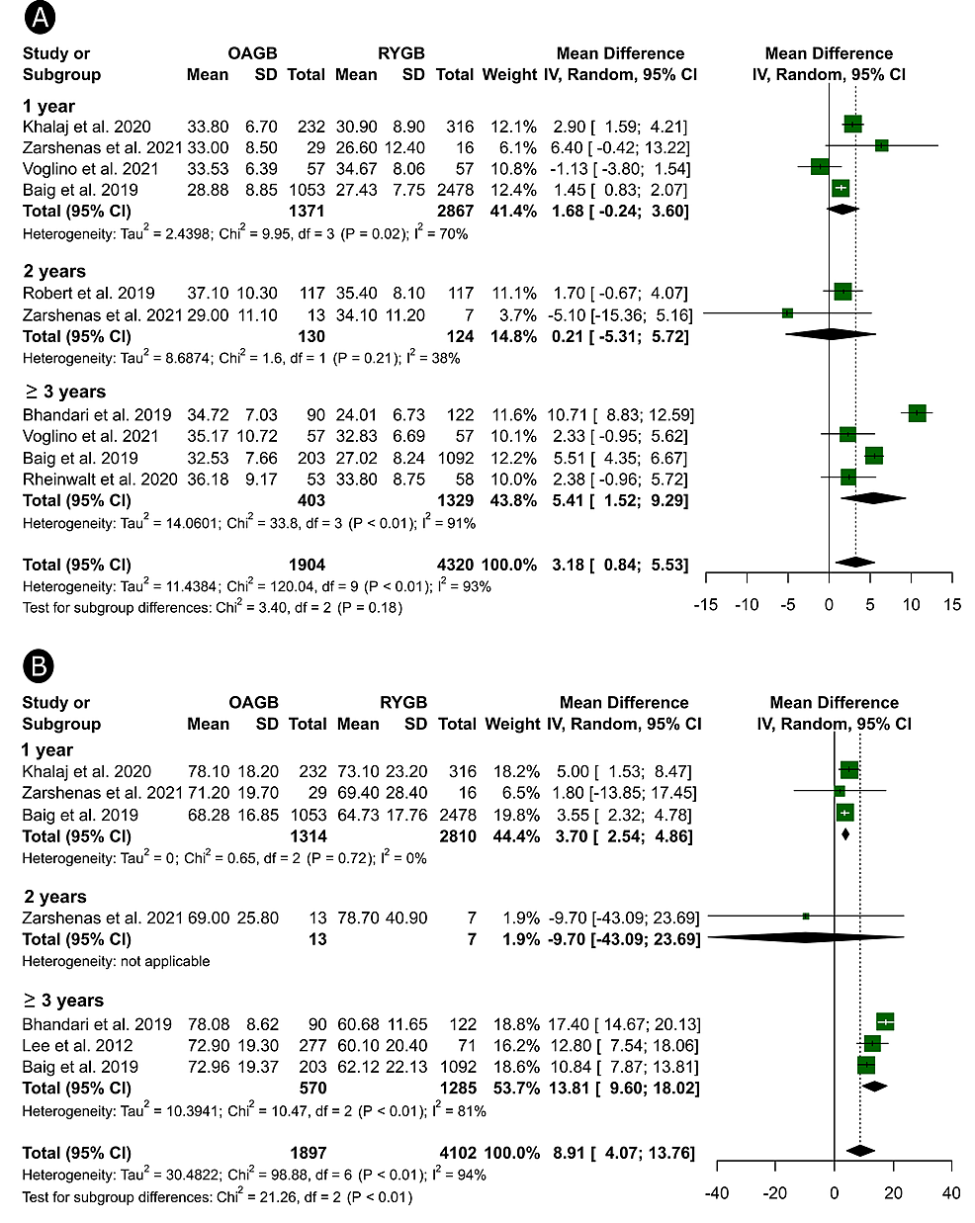
Weight loss differences between OAGB and RYGB OAGB: one-anastomosis gastric bypass; RYGB: Reux-en-Y gastric bypass

At one year of follow-up, patients in the OAGB arm had attained significantly higher %EWL than those in the RYGB arm (MD = 3.70%, 95%CI, 2.54 to 4.86) with no between-group differences in %TWL. However, at ≥ 3 years (the latest follow-up time point), the %TWL and %EWL were significantly higher after OAGB compared to RYGB (MD = 5.41%, 95%CI, 1.52 to 9.29 for %TWL and MD = 13.81%, 95%CI, 9.60 to 18.02 for %EWL, Figure 2).

Primary Outcomes

Table 3 demonstrates a summary of the primary outcomes. In general, malnutrition was significantly associated with OAGB procedures (OR = 3.00, 95%CI, 1.68 to 5.36, p < 0.0001). Robert et al. [17]. have defined malnutrition as albumin concentration of <30 g/L or prealbumin concentration of <0.2 g/L or both, whereas malnutrition was considered as deficiency of total protein, albumin, calcium, iron, or vitamins in the study of Rheinwalt et al. [43]. OAGB procedures were also associated with significantly higher odds of hypoalbuminemia (OR = 2.38, 95%CI, 1.65 to 3.43, p < 0.0001), hypoproteinemia (OR = 1.85, 95%CI, 1.09 to 3.14, p = 0.022), anemia (OR = 1.38, 95%CI, 1.08 to 1.77, p = 0.011), and hypocalcemia (OR = 1.78, 95%CI, 1.01 to 3.12, p = 0.046). Such significant associations were based on pairwise comparisons with no significant heterogeneity (I2 = 0-31%). The odds of vitamin D and vitamin B12 deficiencies, as well as the deficiencies of iron, ferritin, and zinc, were not significantly different between both the laparoscopic procedures.

**Table 3:** Effect sizes and between-study heterogeneity outcomes for nutritional deficiencies after OAGB and RYGB procedures N: number; OAGB: one-anastomosis gastric bypass; RYGB: Roux-en-Y gastric bypass

Parameter	N of studies	N OAGB	N RYGB	Treatment Effect		Heterogeneity		
				OR (95%CI)	p	Model	I^2^(%)	p_h_
Malnutrition	4	3246	1609	3.00 (1.68 to 5.36)	< 0.0001	F	0	0.548
Anemia	9	2836	2504	1.38 (1.08 to 1.77)	0.011	F	0	0.765
Hypoalbuminemia	8	1984	1978	2.38 (1.65 to 3.43)	< 0.0001	F	0	0.458
Hypoproteinemia	4	1160	527	1.85 (1.09 to 3.14)	0.022	F	0	0.542
Hypocalcemia	4	284	214	1.78 (1.01 to 3.12)	0.046	F	31.0	0.226
Vitamin D deficiency	4	282	208	1.29 (0.81 to 2.05)	0.291	F	28.9	0.239
Vitamin B12 deficiency	4	252	194	1.13 (0.36 to 3.58)	0.831	R	62.1	0.048
Iron deficiency	3	194	113	1.26 (0.68 to 2.32)	0.464	F	0	0.844
Ferritin deficiency	3	196	126	1.05 (0.46 to 2.43)	0.902	R	52.2	0.124
Zinc deficiency	2	403	144	1.00 (0.61 to 1.63)	0.989	F	22.8	0.255

Subgroup Analysis

**Figure 3:**
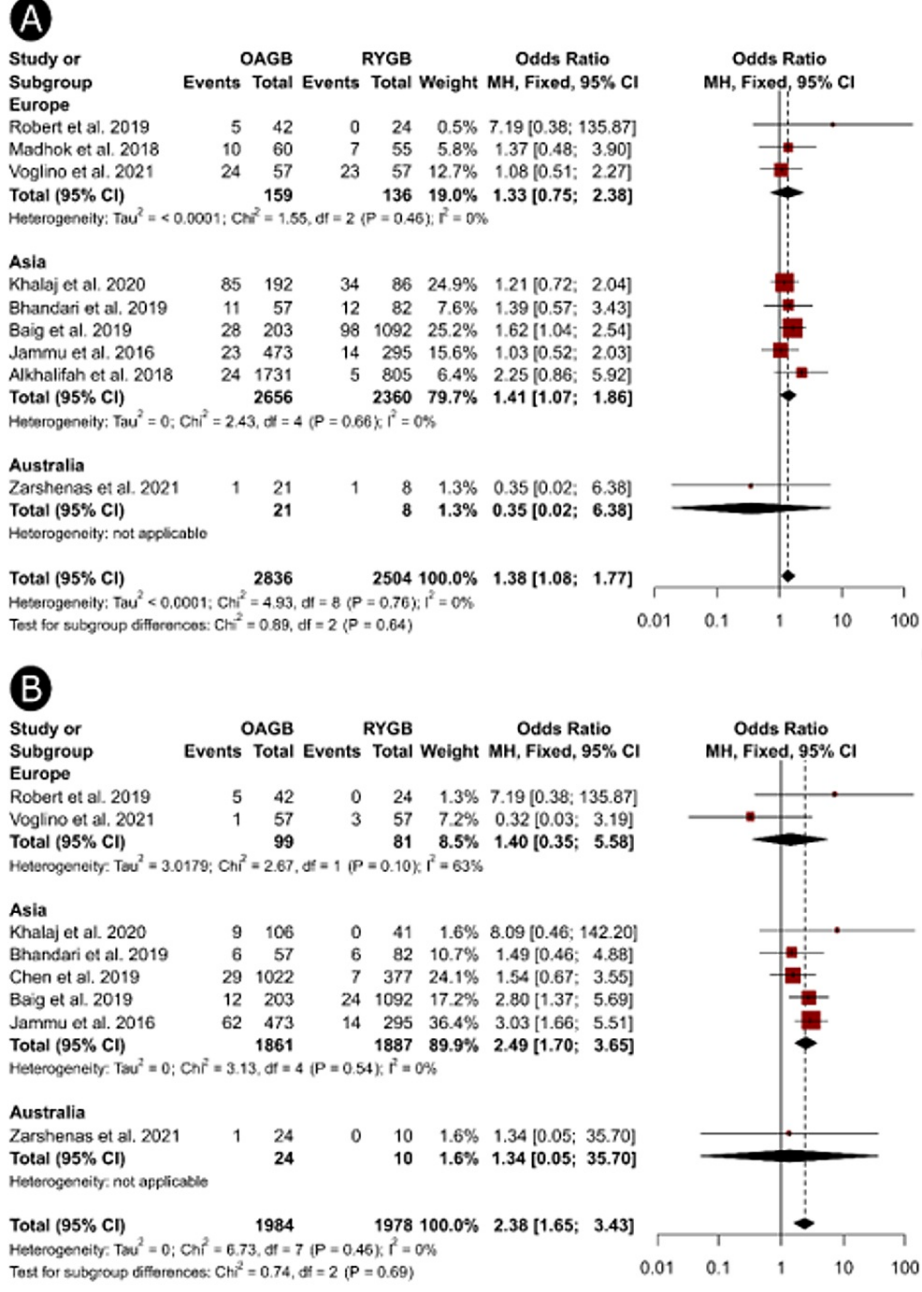
Forest plots showing the results of subgroup analysis based on the study location to investigate the differences in the incidence of anemia (A) and hypoalbuminemia (B) after OAGB and RYGB procedures OAGB: one-anastomosis gastric bypass; RYGB: Reux-en-Y gastric bypass

**Figure 4:**
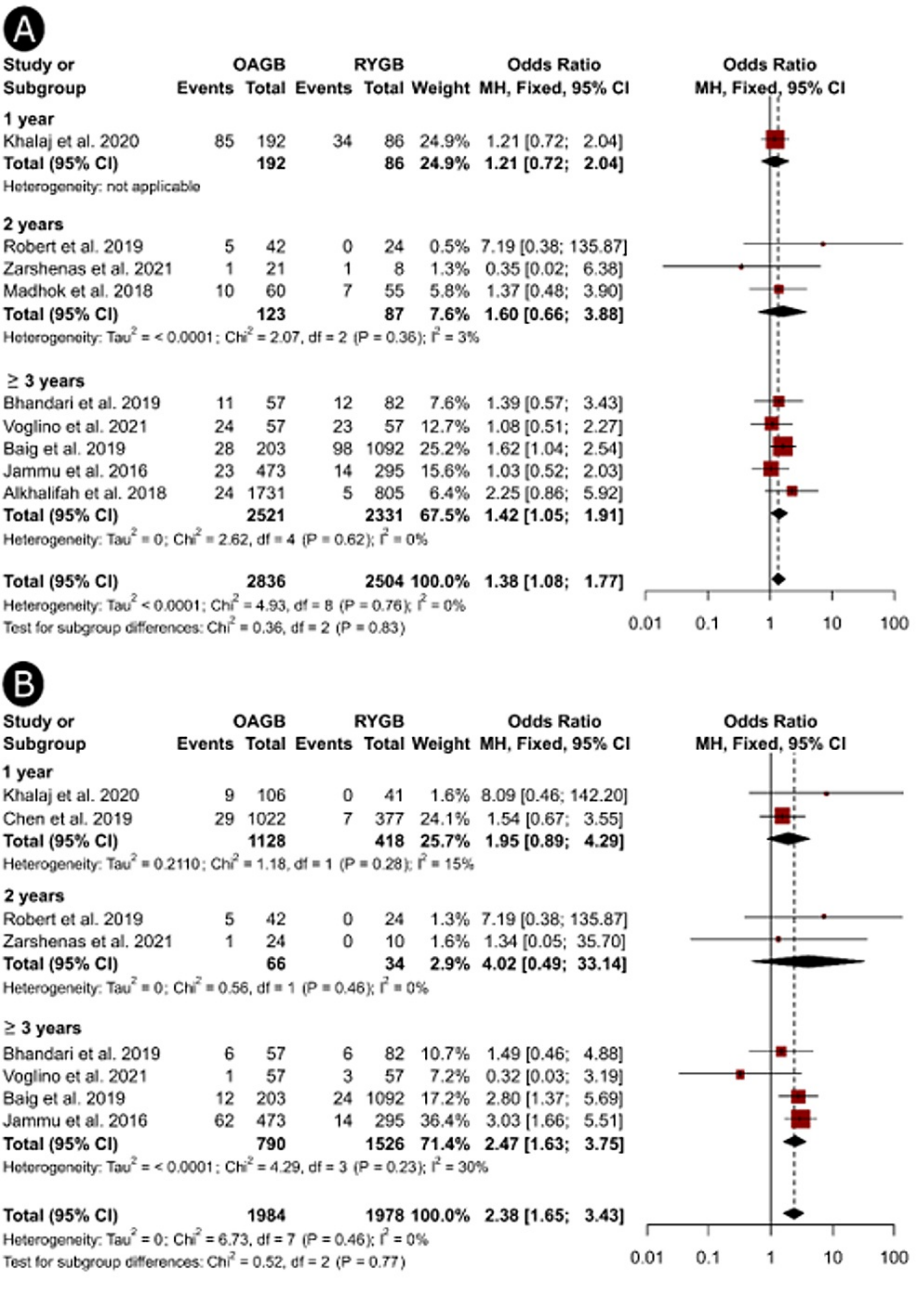
Forest plots showing the results of subgroup analysis based on different follow-up periods to investigate the differences in the incidence of anemia (A) and hypoalbuminemia (B) after OAGB and RYGB procedures OAGB: one-anastomosis gastric bypass; RYGB: Reux-en-Y gastric bypass

On subgroup analysis, we found that studies published in Asia showed significantly higher odds of anemia (OR = 1.41, 95%CI, 1.07 to 1.86, Figure 3A) and hypoalbuminemia (OR = 2.49, 95%CI, 1.70 to 3.65, Figure 3B) among patients who underwent OAGB compared to RYGB; these differences were not evident in the studies published in European countries. Subgroup analysis based on follow-up periods showed significantly higher proportions of anemia (OR = 1.42, 95%CI, 1.05 to 1.91, Figure 4A) and hypoalbuminemia (OR = 2.47, 95%CI, 1.63 to 3.75, Figure 4B) at ≥ 3 years but not at shorter follow-up periods. Additionally, the odds of anemia remained significantly higher after OAGB in studies that recruited >1500 patients (OR = 1.75, 95%CI, 1.17 to 2.62, Figure 6A), whereas medium-sized (OR = 2.44, 95%CI, 1.50 to 3.96) and large-sized studies (OR = 2.80, 95%CI, 1.37 to 5.69, Figure 6B) showed significantly higher proportions of hypoalbuminemia after OAGB. We could not perform a subgroup analysis based on the study design because only one study employed a randomized design [17].

Publication Bias

**Figure 5:**
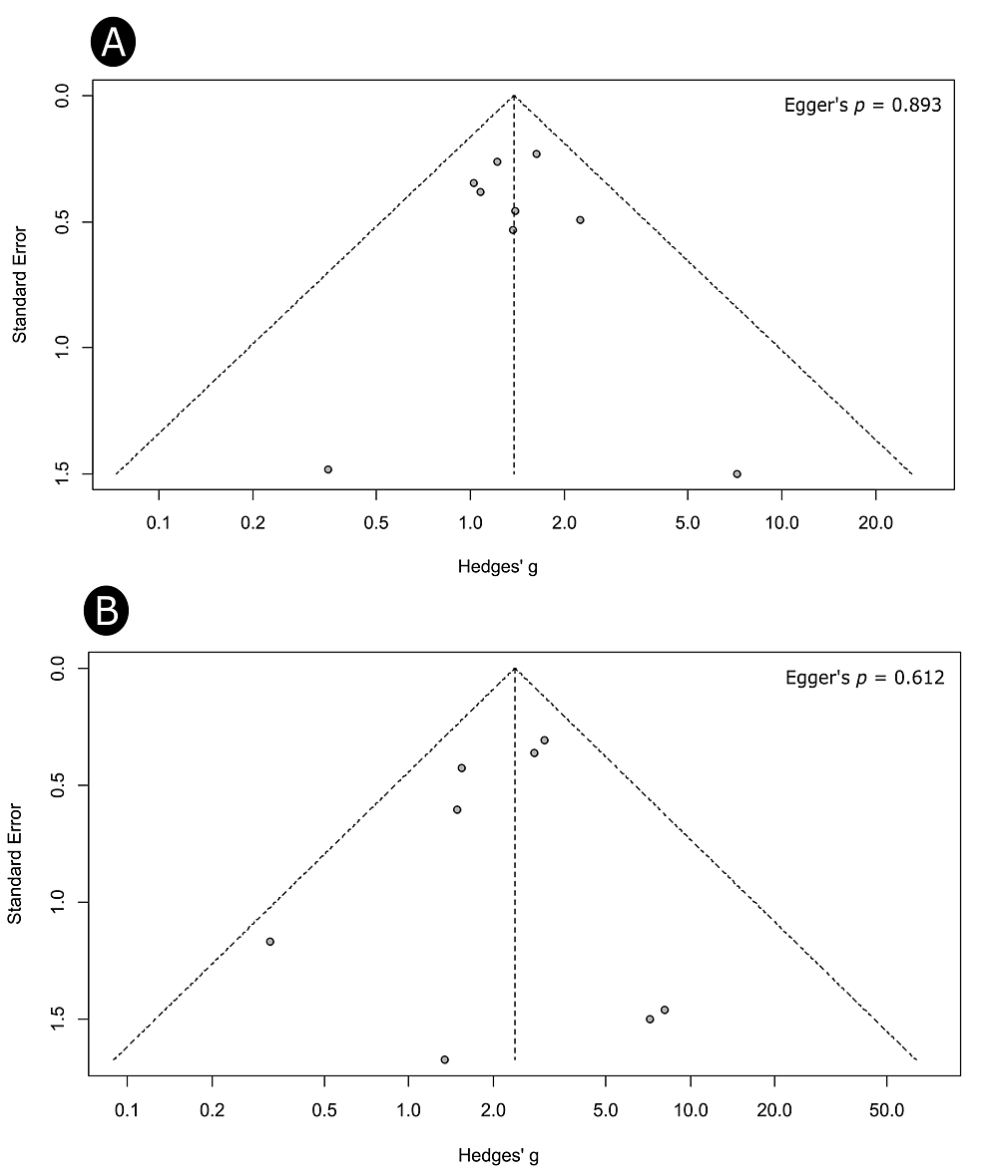
Publication bias

Publication bias was assessed for the outcomes with k > 5 studies. Results revealed that treatment effects were symmetrically distributed around the pooled estimate of anemia (Figure 5A) and hypoalbuminemia (Figure 5B). The outcomes of Egger’s test have also indicated no risk of publication bias (intercept = 0.09, p = 0.893 for anemia and intercept = -0.37, p = 0.612 for hypoalbuminemia).

Discussion

Postoperative morbidity remains a significant challenge for bariatric surgeries. The malabsorptive effect of these surgeries may pose negative nutritional consequences. In the present meta-analysis, we found that OAGB procedures were associated with malnutrition and anemia, as well as deficiencies of blood albumin, total proteins, and calcium.

To the best of our knowledge, there are two published meta-analyses comparing OAGB and RYGB procedures [46,47]; those studies were primarily focused on procedural characteristics, as well as weight loss and metabolic outcomes. Although the previous meta-analyses showed that OAGB was associated with malnutrition, which is consistent with our meta-analysis, the authors did not investigate additional nutritional outcomes. With the inconsistent definition of malnutrition across the included studies, it was difficult to conclude reliable outcomes that could be further considered in the postoperative periods. Therefore, in the present study, we have heavily focused on specific parameters that could help investigate the potential sources of nutritional deficiencies to improve the standards of efficiency and safety of gastric bypass surgeries.

As with the aforementioned studies [46,47], we showed that patients in the OAGB arm could achieve better weight loss than RYGB. Although OAGB includes making a long sleeve tube and RYGB entails creating a small gastric pouch and small outlet, the gastric restriction effect is generally similar for both the procedure. Furthermore, the pattern of gut hormonal response is similar, where duodenal gut hormones and related enzymes are excluded by the duodenojejunal bypass, whereas a postprandial surge of distal gut hormones (glucagon-like peptide 1 (GLP-1) and peptide YY (PYY)) is observed in both procedures [48,49]. As such, weight-related differences can solely be explained by the variation in the bypass limb length. The standard biliopancreatic limbs (BPLs) in RYGB and OAGB surgeries are 50 and 200 cm, respectively. There is now accumulated evidence indicating that a longer BPL is associated with a better weight loss and more favorable glycemic control [50,51]; yet, better efficacy and safety profiles could be achieved for the effect of revision surgery at a threshold of < 70% of the small bowel [52].

Seemingly, BPL might have important roles in the observed nutritional deficiencies. For instance, Jammu et al, [38]. found that hypoalbuminemia was significantly more frequent among patients with a limb length of >230 cm, while only one case was reported among those with a 200 cm BPL. Mahawar et al. [53] have also suggested that a BPL of 150 cm is recommended in OAGB procedures to reduce the likelihood of severe protein-calorie malnutrition. Unfortunately, we could not assess the role of BPLs on the development of nutritional complications due to the lack of standardized lengths in the included studies (Table 1).

We believe technical modifications of gastric bypass procedures may also play a significant role in changing the risk profile of nutritional deficiencies, and this should be a matter of in-depth investigation to reach the standardized lengths of the small bowel bypassed that achieve maximum weight loss with minimum nutritional deficiencies.

Notably, other reasons may influence protein absorption in OAGB. Supposedly, hypoalbuminemia and hypoproteinemia are more pronounced in OAGB as a result of proximal jejunal bypass. Elgeidie et al. [54] proposed that protein-energy undernutrition may develop due to protein intolerance (an imbalance in bacterial flora, reduced pepsin production, and changes in gut hormones), steatorrhea (pancreatic proteolytic enzymes are deactivated in the acidic pH of the gastric pouch), postoperative incompliance to supplementation and ethnic variations. Interestingly, the effect of ethnicity on protein absorption was prominent in our study, with higher odds of hypoalbuminemia after OAGB in studies conducted on patients from Asia but not other continents. On the other hand, it has been previously shown that the absorption of meal protein-derived amino acids is accelerated after RYGB [55], which might partly explain the reported difference in our study.

Regarding other nutritional consequences, we found that OAGB was associated with hypocalcemia, and this might be related to the exclusion of a significant proportion of the proximal small bowel (the site of calcium absorption) [56]. The rates of anemia were significantly higher in the OAGB arm due to the reduced capacity to absorb iron. However, we could not find significant differences between OAGB and RYGB in the rates of iron deficiency; the small number of studies in such a comparison might have contributed to this finding.

Limitations

In the present study, there are several limitations that should be appraised. The reported outcomes were retrieved from retrospective studies and one open-label RCT. This might interfere with concluding reliable results and limit our understanding of the causal relationships between bariatric surgeries and nutritional complications. Also, given the lack of published studies in certain areas, such as the United States, we could not comprehensively assess postoperative complications across different ethnic groups. Finally, the small number of included studies might have impacted the results of some nutrients (iron, ferritin, and zinc) and for studies with long follow-up periods (≥3 years).

## Conclusions

In conclusion, while OAGB surgery induced significantly higher weight loss than RYGB as indicated by %EWL and %TWL, OAGB procedures were associated with multiple nutritional deficiencies, including hypoalbuminemia, hypoproteinemia, and hypocalcemia. Additionally, higher proportions of anemia and malnutrition were observed after OAGB compared to RYGB. The results should be interpreted with caution given the inherent limitations of studies’ design (primarily retrospective investigations) and the small number of studies that assessed nutritional differences over long follow-up periods. Future large-sized RCTs are required to assess the efficacy and safety of OAGB on weight loss and nutritional outcomes, considering the roles of BPL length, ethnic variation, gut hormonal response, and the malabsorptive paradigm of gastric bypass surgeries.
